# Proteostasis-targeted antibacterial strategies

**DOI:** 10.71150/jm.2511007

**Published:** 2026-02-12

**Authors:** Yoon Chae Jeong, Seong-Hyeon Kim, Seongjoon Moon, Hyunhee Kim, Changhan Lee

**Affiliations:** 1 Department of Biological Sciences, Ajou University, Suwon 16499, Republic of Korea; 2 Ajou Energy Science Research Center, Ajou University, Suwon 16499, Republic of Korea; 3 Research Institute of Basic Sciences, Ajou University, Suwon 16499, Republic of Korea

**Keywords:** proteostasis, protein quality control system, chaperone, protease, BacPROTAC, APP, persister, VBNC

## Abstract

Protein quality control systems are increasingly recognized as a critical determinant of bacterial survival and antibiotic tolerance. Conventional antibiotics predominantly target nucleic acids, protein synthesis, or cell wall synthesis, yet bacterial adaptation and resistance emergence remain major challenges. Targeting the bacterial protein quality control machineries including molecular chaperones and proteases offers a promising strategy to overcome these limitations. Recent advances include small molecules and adaptor/degron mimetics that modulate the activities of chaperones and proteases, aggregation-prone peptides (APPs) that induce proteotoxic stress, and bacterial PROTAC (BacPROTAC) strategies that redirect endogenous proteases. Notably, persister and viable-but-non-culturable (VBNC) cells, which tolerate conventional antibiotics, remain susceptible to proteostasis-targeted approaches, thereby enabling killing in both actively dividing and dormant populations. Furthermore, synergistic strategies combining chaperone inhibition or protease activation with conventional antibiotics enhance bactericidal efficacy, suggesting a potential avenue to mitigate antimicrobial resistance. This review summarizes the mechanistic basis, recent developments, and translational potential of proteostasis-centered antibacterial strategies.

## Introduction

Antibiotic resistance represents a critical global health threat that undermines decades of success in controlling infectious diseases (Chinemerem Nwobodo et al., 2022; Edgar et al., 2008; Klein et al., 2024). Classical antibacterial agents have been developed to exploit bacterial-specific macromolecular processes, including nucleic acid synthesis, cell wall biosynthesis, membrane integrity, and protein synthesis (Baran et al., 2023; Butler et al., 2024; Halawa et al., 2024; Kohanski et al., 2010; O’Rourke et al., 2020). Despite their success, these single-target and site-specific mechanisms provide short evolutionary routes to resistance. Examples include *rpoB* point mutations conferring rifampicin resistance, *gyrA*/*parC* substitutions or *qnr*-mediated protection against fluoroquinolones, penicillin-binding protein remodeling and β-lactamase activity defeating β-lactams, and rRNA methyltransferases or aminoglycoside-modifying enzymes inactivating aminoglycosides (Belay et al., 2024; Goldstein, 2014; Hooper, 2001; Serio et al., 2018; Teichmann et al., 2025; Zapun et al., 2008). As a result, multidrug-resistant (MDR), extensively drug-resistant (XDR), and tolerant bacterial phenotypes—including persisters and viable-but-non-culturable (VBNC) states—have become increasingly prevalent (Almutairy, 2024; Bharadwaj et al., 2022; Michiels et al., 2016; Niu et al., 2024).

Proteins are the main functional components of the bacterial cell, and their synthesis, folding, and degradation are governed by a conserved proteostasis network (Koga et al., 2011). This network heavily relies on ATP-dependent chaperones such as DnaK/Hsp70, GroEL/ES, and ClpB to assist folding and prevent aggregation (Balchin et al., 2016), as well as AAA+ (ATPases associated with diverse cellular activities) proteases such as Lon, FtsH, and ClpXP/ClpAP to remove misfolded or regulatory proteins through adaptor-mediated recognition (Dalbey et al., 2012; Gottesman et al., 1998; Gur et al., 2012; Sauer and Baker, 2011). Conventional translation inhibitors paradoxically increase the cellular burden of defective polypeptides, straining this proteostasis network. When chaperone buffering or proteolytic clearance fails, misfolded proteins accumulate, leading to toxic aggregation and proteome collapse (Khodaparast et al., 2021; Pytel and Fromm Longo, 2025). This systems-level fragility highlights proteostasis as a potential vulnerability that can be pharmacologically exploited.

Recently, antimicrobial strategies that directly perturb bacterial proteostasis have gained attention. These include small molecules targeting chaperones or proteases to compromise quality control capacity, and aggregation-prone antimicrobial peptides that disrupt protein homeostasis (Khodaparast et al., 2018, 2021). Notably, proteostasis-targeted strategies have shown efficacy against phenotypically tolerant populations such as persisters and VBNC cells—populations often refractory to conventional antibiotics targeting DNA, the cell wall, or ribosomes. In this review, we summarize the architecture and stress-responsive dynamics of bacterial proteostasis, discuss recent advances in chemical and genetic modulation of chaperones and proteases, and explore the potential of proteostasis-targeted therapeutics as a next-generation strategy to overcome bacterial tolerance and resistance to antibiotics.

## A Proteostasis-Centered View of Protein Life Cycle

This section focuses on how proteostasis governs the bacterial protein life cycle and its relevance to antibiotic action. Proteostasis, or protein homeostasis, is a central principle of cellular physiology that ensures proteins maintain their correct structure and function. It encompasses all stages of the protein life cycle—from synthesis and folding to degradation—coordinated by chaperones, proteases, and regulatory cofactors to prevent the accumulation of misfolded or aggregated proteins.

Proteins synthesized by ribosomes must fold correctly to function. Small proteins often fold spontaneously, but most bacterial proteins require assistance due to their complex structures and aggregation tendency (Balchin et al., 2016; Khodaparast et al., 2021). In vivo, this process is challenged by macromolecular crowding, folding intermediates, and environmental stress. Bacteria, lacking compartmentalized quality control systems, rely heavily on molecular chaperones and proteases to maintain proteostasis and regulate protein abundance and folding efficiency. The bacterial 70S ribosome orchestrates translation through initiation, elongation, and termination, assisted by elongation factors and ribosome-associated chaperones (Balchin et al., 2016; Deuerling et al., 2019; Wilson, 2014). Nascent chains exit through the 50S tunnel, where limited space restricts folding (Wilson and Beckmann, 2011). Translational pauses and co-translational interactions with trigger factor and other chaperones facilitate proper domain formation and prevent premature aggregation (Cassaignau et al., 2020; Samatova et al., 2021).

**Fig. 1.**
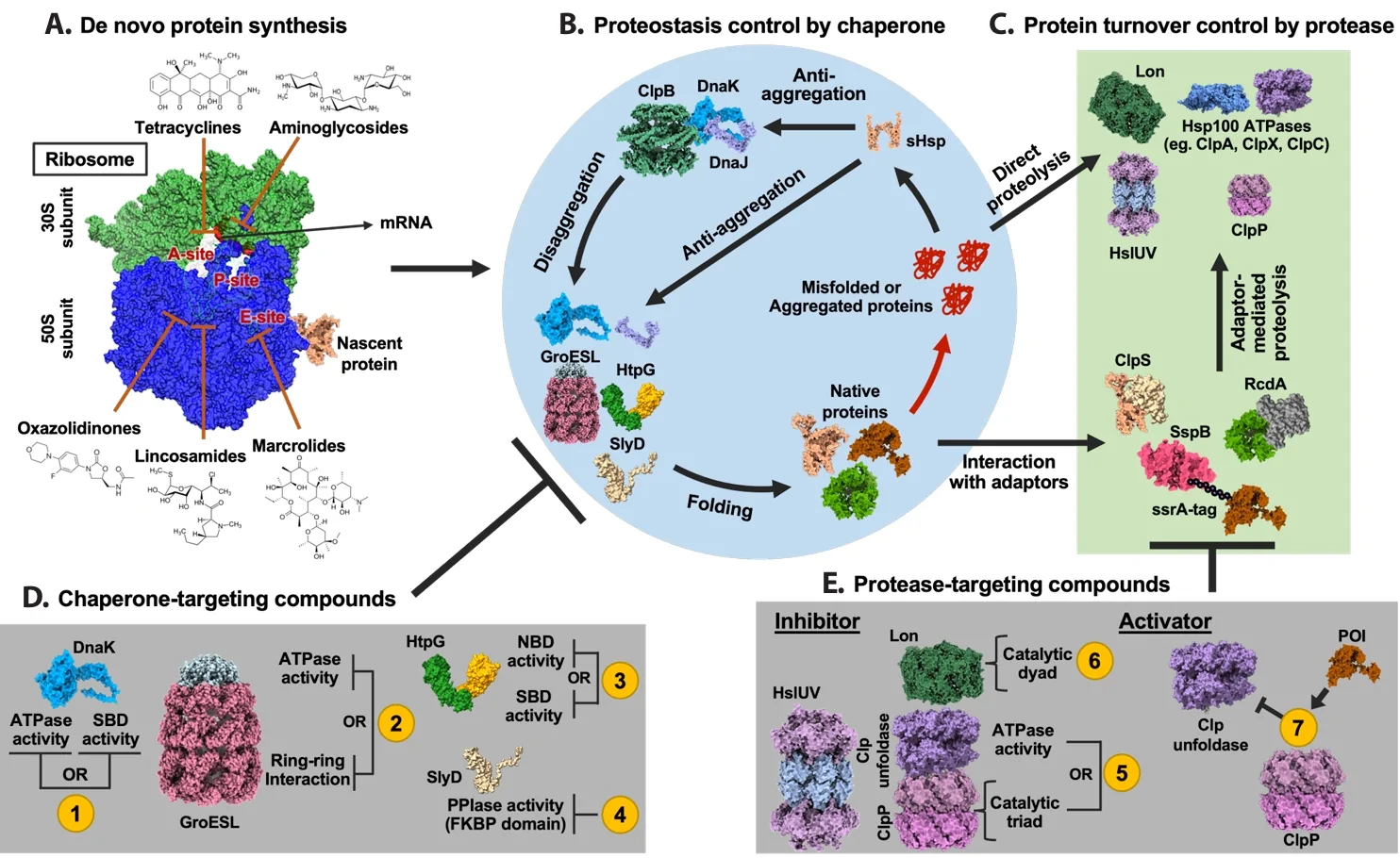
Overview of antibiotics targeting proteostasis in bacterial cells. From a proteostasis-centered perspective on the life cycle of a protein, (A) existing antibiotics disrupt proteostasis in nascent polypeptides emerging from the ribosome by causing protein misfolding and aggregation. (B, C) The chaperone and protease systems, which control protein quality, protect cells from the effects of antibiotics by restoring disturbed proteostasis. (D) Antibiotics targeting chaperones inhibit their functions in maintaining proteostasis. In addition, (E) antibiotics targeting proteases not only inhibit proteolysis but also perturb the regulation of proteolytic activity, leading to indiscriminate protein degradation. The indicated numbers in (D, E) represent drugs that targeting chaperone or protease, and listed in Table 1 (SBD: substrate binding domain, NBD: nucleotide binding domain, POI: protein of interest).

After release from the ribosome, molecular chaperones—such as DnaK/DnaJ/GrpE, GroEL/ES, and trigger factor—assist nascent polypeptides in achieving their native conformations (Fig. 1) (Mogk et al., 2011). When folding is perturbed, holdases like small heat shock proteins transiently stabilize misfolded intermediates, whereas disaggregases such as ClpB cooperate with DnaK to resolubilize protein aggregates (Bohl and Mogk, 2024; Obuchowski et al., 2021). Together, these ATP-dependent and ATP-independent machineries form a dynamic proteostasis network that safeguards the bacterial proteome under stress. When refolding fails, damaged or terminally misfolded proteins are eliminated by proteolysis. AAA+ proteases, including ClpAP, ClpXP, and Lon, recognize and degrade aberrant substrates through ATP-driven unfolding and proteolysis, while adaptor proteins such as ClpS and RcdA refine substrate specificity and couple degradation to stress adaptation (Kuhlmann and Chien, 2017; Mahmoud and Chien, 2018). The coordinated interplay between chaperones and proteases thus maintains a delicate balance between folding, refolding, and degradation to sustain bacterial proteostasis.

## Antibiotic Effects on Bacterial Proteostasis

Ribosome-targeting antibiotics directly interfere with translation, inducing translational errors, misfolded proteins, and aggregation, which challenge bacterial proteostasis networks (Fig. 1A) (Kohanski et al., 2010; Thompson et al., 2002; Wilson, 2014). Tetracyclines bind to the ribosomal A site, blocking tRNA binding and initiating stress responses that elevate chaperone expression including DnaK, GroEL, and ClpB (Møller et al., 2020). Aminoglycosides, by inducing mistranslation and aggregation, directly trigger proteotoxic stress, which is mitigated by chaperone systems (Goltermann et al., 2013; Thompson et al., 2002). Similarly, oxazolidinones and lincosamides inhibit peptide bond formation at the peptidyl transferase center, while macrolides stall translation within the exit tunnel (Kapoor et al., 2017; Liang et al., 2012; Thompson et al., 2002). Although their mechanisms differ, these antibiotics converge in eliciting a chaperone-mediated stress response to restore protein homeostasis.

In addition, other antibiotic classes indirectly disrupt proteostasis by modifying the cytoplasmic environment. β-Lactams disrupt peptidoglycan crosslinking, alter osmotic balance, and impair energy metabolism (Lobritz et al., 2022; Wong et al., 2021), while fluoroquinolones and aminoglycosides promote reactive oxygen species accumulation (Guillouzo and Guguen-Guillouzo, 2020; Kohanski et al., 2008), thereby exacerbating proteotoxic stress. With accordance, various molecular chaperones are induced upon various stresses including osmotic shock, membrane damage and oxidative stresses (Bojanovič et al., 2017; Dawan and Ahn, 2022; Storey and Storey, 2023). These results suggest that protein quality control system is involved as a universal bacterial defense mechanism regardless of antibiotic class.

Given the universal involvement of response to antibiotic treatments, targeting bacterial proteostasis itself offers a promising therapeutic direction and several molecules has been developed (Fig. 1B–1E, Table 1). Among, various chaperones, DnaK, GroEL/ES, HtpG, and SlyD have emerged as attractive targets due to their essential roles in folding and aggregation control (Abdeen et al., 2016; Carlson et al., 2024; Chiappori et al., 2015; Kumar and Balbach, 2017). Small-molecule inhibitors—including Telaprevir, PET-16, and BI88E3—disrupt DnaK activity by interfering with nucleotide or substrate binding (Cellitti et al., 2009; Hosfelt et al., 2022; Leu et al., 2014). Similarly, GroEL/ES and HtpG inhibitors block ATPase cycling and substrate folding, while metal complexes targeting SlyD inhibit both PPIase and chaperone function, leading to growth inhibition in diverse pathogens (Carlson et al., 2024; Kumar and Balbach, 2017; Wang et al., 2025). Collectively, inhibitors that collapse bacterial proteostasis represent a mechanistically distinct and potentially resistance-resilient approach to antibiotic development. We will first discuss the role of molecular chaperones in antibiotic responses and strategies for targeting chaperones, and then move on to targeting proteases.

## Importance of Chaperones in Antibiotic Effects

Disruption of chaperone function has been shown to increase antibiotic sensitivity and reduce bacterial resistance (Hosfelt et al., 2022; Lukačišinová et al., 2020; Richards and Lupoli, 2023). This section primarily discusses antibiotics that interfere with protein synthesis and how bacterial chaperone systems counteract their effects. Notably, several key chaperone networks—including the DnaK/DnaJ/GrpE system, GroEL/ES chaperonins, and the ClpB disaggregase—play pivotal roles in mitigating antibiotic-induced proteotoxicity.

### DnaK/DnaJ/GrpE system

The DnaK/DnaJ/GrpE chaperone system constitutes the central hub of bacterial proteostasis, functioning as the bacterial equivalent of the Hsp70 machinery in eukaryotes. It cooperates with GroEL/ES chaperonins, ClpB disaggregase, small heat shock proteins, and proteolytic complexes to manage misfolded proteins and maintain protein homeostasis under stress (Cho et al., 2024; Rosenzweig et al., 2013). Among these, DnaK plays a particularly critical role during antibiotic exposure, where proteotoxicity is induced by drug-driven mistranslation and misfolding (Fay et al., 2021; Goltermann et al., 2013; Ling et al., 2012; Richards and Lupoli, 2023).

Mutational disruption of *dnaK* markedly affects bacterial fitness and antibiotic resistance. Aminoglycosides such as streptomycin, kanamycin, and gentamicin exemplify induce accumulation of misfolded proteins, resulting in upregulation of chaperone genes, including *dnaK*, *dnaJ*, *grpE*, and *groEL*/*ES* (Goltermann et al., 2013; Ling et al., 2012). Overexpression of the *dnaK*/*dnaJ*/*grpE* system in *E. coli* improves bacterial survival in the presence of aminoglycosides, while *dnaK* mutants show impaired growth and heightened cell death due to proteotoxic aggregation (Fay et al., 2021; Goltermann et al., 2013; Hosfelt et al., 2022; Richards and Lupoli, 2023).

The DnaK system also modulates resistance to rifampicin, an RNA polymerase inhibitor widely used in tuberculosis treatment (Stefan et al., 2018; Telenti et al., 1993). In mycobacteria, mutations in *rpoB*, encoding the β-subunit of RNA polymerase, confer rifampicin resistance but simultaneously destabilize the enzyme (Fay et al., 2021). The DnaK/DnaJ machinery mitigates this instability by stabilizing mutant RpoB proteins. *Mycobacterium* strains lacking DnaK or DnaJ2 exhibit severe fitness defects and loss of viability in the presence of rifampicin (Fay et al., 2021). Furthermore, DnaK levels influence the frequency of resistance development—higher DnaK expression promotes resistance acquisition, whereas lower expression reduces resistance frequency up to sixfold, likely by increasing proteotoxic stress (Fay et al., 2021).

Similar findings have been reported in *Campylobacter jejuni*, where Δ*dnaK* mutants exhibit reduced viability and compromised antibiotic resistance following exposure to tetracycline and ciprofloxacin which targets protein synthesis and DNA synthesis, respectively (Cho et al., 2024). Transmission electron microscopy confirmed the accumulation of protein aggregates in *dnaK* mutants under antibiotic stress (Cho et al., 2024). Collectively, these findings underscore that the DnaK/DnaJ/GrpE system not only preserves bacterial proteostasis but also shapes antibiotic sensitivity and the evolution of resistance.

### GroEL/ES chaperonins

The GroEL/ES chaperonin complex functions in conjunction with DnaK/DnaJ/GrpE to ensure correct protein folding and prevent aggregation, especially during antibiotic stress (Goltermann et al., 2013, 2016). Inhibition or deletion of *groEL*/*ES* markedly increases bacterial sensitivity to antibiotics, impairs cytosolic protein folding, and causes severe growth defects in the presence of aminoglycosides such as streptomycin and gentamicin, likely due to disruption of membrane potential (Goltermann et al., 2013). Conversely, GroEL/ES overexpression enhances survival by restoring membrane integrity, reducing protein misfolding, and improving growth even more effectively than DnaK overexpression (Goltermann et al., 2013, 2016). In *C. jejuni*, Δ*groEL*/*ES* strains show increased sensitivity to tetracycline and ciprofloxacin, exhibiting extensive cell death under tetracycline treatment (Cho et al., 2024). These findings suggest that the GroEL/ES system acts as a secondary defense line against antibiotic-induced cytotoxicity, facilitating bacterial adaptation and tolerance (Cho et al., 2024; Godek et al., 2024; Wang et al., 2025).

Given its central role in maintaining proteome integrity, the GroEL/ES system has gained attention as a potential drug target. Several studies have demonstrated that its inhibition disrupts essential folding processes, leading to bacterial death in various pathogens (Cho et al., 2024; Goltermann et al., 2013, 2016). Furthermore, small molecules targeting GroEL/ES have shown potent antimicrobial activity, highlighting its potential as a target for combating multidrug-resistant bacteria (Abdeen et al., 2018; Godek et al., 2024; Wang et al., 2025). Thus, GroEL/ES not only supports bacterial survival under proteotoxic stress but also contributes to the persistence and evolution of antibiotic tolerance.

### ClpB disaggregase

Under antibiotic stress, bacterial proteins often misfold and aggregate into inclusion bodies. The ClpB disaggregase, a member of the Hsp100 family, cooperates with DnaK to resolubilize and refold these aggregated proteins, forming a bichaperone complex critical for stress recovery (Calloni et al., 2012; Mogk, 1999). Several studies have linked ClpB function to antibiotic resistance. Δ*clpB* mutants are highly sensitive to multiple antibiotics and exhibit reduced capacity to recover from proteotoxic conditions (Calloni et al., 2012; Cho et al., 2024; Harnagel et al., 2021). In *E. coli*, exposure to kanamycin upregulates *clpB* expression alongside other chaperones involved in protein refolding (Calloni et al., 2012). In *C. jejuni*, Δ*clpB* mutants accumulate aggregated proteins and show severe growth inhibition under tetracycline treatment (Cho et al., 2024). Similarly, in *Mycobacterium tuberculosis*, ClpB facilitates survival during antibiotic stress by sequestering toxic aggregates asymmetrically, thereby protecting daughter cells from proteotoxic overload (Vaubourgeix et al., 2015). Loss of ClpB increases susceptibility to kanamycin, streptomycin, and geldanamycin (Harnagel et al., 2021; Vaubourgeix et al., 2015). Together, these observations emphasize that ClpB-mediated disaggregation is indispensable for bacterial persistence under antibiotic-induced proteotoxic stress. By coupling with DnaK, ClpB ensures proteome renewal and cellular recovery—functions that make it an appealing target for novel antimicrobial strategies aimed at collapsing bacterial proteostasis networks.

## Potentiation of Antibiotics by Chaperone Inhibition

Disrupting chaperone function can sensitize bacteria to conventional antibiotics, offering a rational strategy for combination therapy. Here, we present examples of antibiotic use in combination with chaperone inhibition (Table 2).

Inhibition of DnaK has been shown to sensitize bacteria to proteotoxic antibiotics. For instance, Telaprevir, originally developed as a protease inhibitor, suppresses cofactor-mediated activation of DnaK in *Mycobacterium* species, leading to enhanced susceptibility to aminoglycosides, such as kanamycin and streptomycin, under heat or proteotoxic stress (Hosfelt et al., 2022). Sublethal Telaprevir does reduce minimal inhibitory concentration and decrease the frequency of rifampicin-resistant mutants, demonstrating the potential of DnaK inhibition to amplify antibiotic efficacy (Goltermann et al., 2013; Hosfelt et al., 2022). Proline-rich antimicrobial peptides (PrAMPs), including pyrrhocoricin and synthetic dimers, also target DnaK. These peptides bind the chaperone, disrupt protein folding, and synergize with β-lactams or quinolones by accumulating misfolded proteins, which enhances proteotoxic stress and potentiates antibiotic killing (Kragol et al., 2001). Collectively, these studies establish DnaK as a viable target for combination therapy to overcome bacterial stress-buffering mechanisms.

Aminoglycosides, such as gentamicin, induce misfolded protein accumulation in the cytoplasm. Normally, GroEL/ES buffers this stress, limiting the bactericidal effect. Hydroxybiphenylamide derivatives and related small-molecule inhibitors reduce GroEL/ES folding capacity and impair biofilm survival in *S. aureus* (Kunkle et al., 2018). Co-treatment with sublethal doses of these inhibitors enhances aminoglycoside-mediated killing, providing a mechanistic rationale for combination therapy. By limiting chaperone buffering, bacteria are rendered more susceptible to proteotoxic stress, resulting in synergistic bactericidal activity and sensitizing β-lactam-resistant strains. Together, these findings provide a mechanistic rationale for combination strategies targeting bacterial chaperone to potentiate antibiotic action and mitigate resistance.

## Aggregation-Prone Peptides (APPs)

**Fig. 2.**
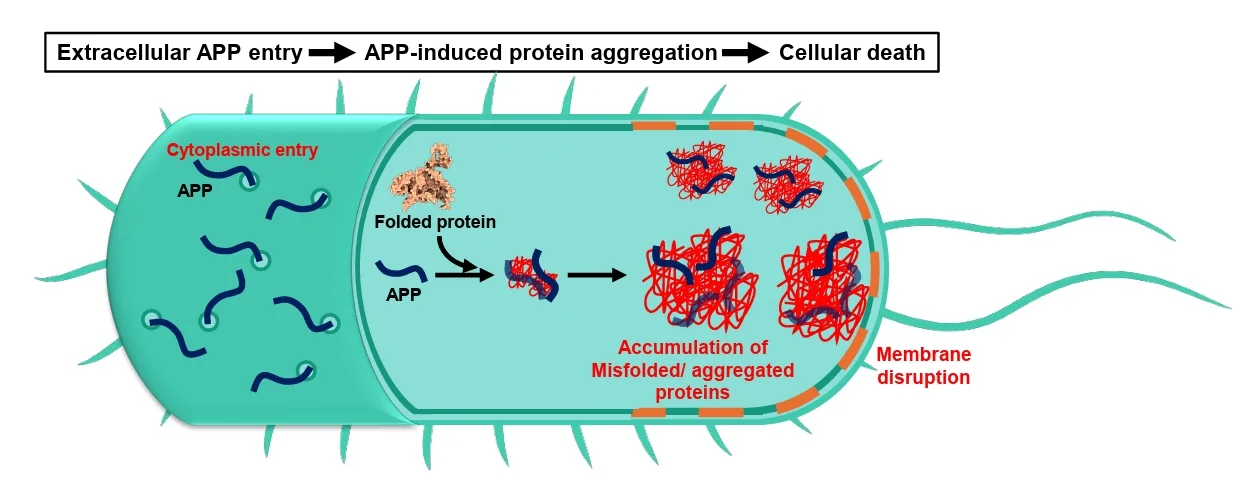
Mechanistic overview of aggregation-prone peptides (APPs) disrupting bacterial proteostasis. APPs enter bacterial cells and interact with nascent or misfolded proteins, promoting aberrant aggregation. The accumulation of aggregates overwhelms chaperone and protease systems, leading to proteostasis collapse and cell death.

Building upon the proteostasis-centered framework discussed above, recent research has explored a novel antibacterial strategy that actively collapses bacterial protein homeostasis. Among these approaches, APPs have emerged as a powerful means to selectively trigger intracellular proteotoxic stress, leading to rapid bacterial killing (Bednarska et al., 2016; De Baets et al., 2015; Morales et al., 2013; Torrent et al., 2011) (Fig. 2).

### Design and mechanistic rationale of APP

APPs are typically designed from 5–7 amino acid-long aggregation-prone regions (APRs) identified through bioinformatic algorithms such as TANGO, which predict aggregation-prone sequences based on physicochemical properties and thermodynamic parameters (Fernandez-Escamilla et al., 2004; Khodaparast et al., 2018). To enhance solubility and prevent premature aggregation during synthesis, the flanking regions are often modified with positively charged residues, such as arginine, which act as aggregation gatekeepers and facilitate bacterial uptake (Hancock and Chapple, 1999; Rousseau et al., 2006). The net positive charge of APPs also promotes interaction with negatively charged bacterial cell envelopes, increasing intracellular delivery efficiency without causing immediate membrane lysis (Bednarska et al., 2016).

Mechanistically, APPs leverage homologous seeding of aggregation in which peptides with sequences identical or highly similar to APRs of essential bacterial proteins efficiently nucleate aggregation, whereas heterologous seeding in mammalian cells is inefficient due to sequence divergence (Ganesan et al., 2015; Khodaparast et al., 2018; Morales et al., 2013). Proteomics analyses of APP-induced inclusion bodies revealed sequestration of hundreds of essential proteins, including metabolic enzymes and chaperones, illustrating a systems-level disruption of proteostasis (Khodaparast et al., 2018). This widespread proteostatic overload results in cellular dysfunction, impaired division, and eventual bacterial death (Bednarska et al., 2016; Lashuel and Lansbury, 2006).

### Sequence-defined aggregation toxicity of APPs

Unlike membrane-active antimicrobial peptides, APPs display sequence-dependent intracellular toxicity. Scrambled peptides with similar hydrophobicity can still aggregate but lack bactericidal potency, demonstrating that precise sequence complementarity to bacterial APRs is the critical determinant of activity (Khodaparast et al., 2018). Thus, the bactericidal activity of APPs arises not from general aggregation, but from sequence-specific molecular recognition within the bacterial proteome. This indicates that APPs do not simply induce nonspecific aggregation; rather, they exploit homologous seeding, where sequence-matched segments trigger co-aggregation with essential bacterial proteins, leading to proteostasis collapse.

Comparative studies suggest that Gram-positive bacteria, such as *S. aureus* and *Enterococcus faecalis*, are more susceptible than Gram-negative species, likely due to cell wall permeability differences and the accessibility of target APRs (Bednarska et al., 2016). Importantly, APPs spare mammalian cells in vitro and in vivo, despite the presence of homologous sequences in the human proteome. This selectivity may be explained by several factors which are inefficient heterologous seeding, differences in cell size and metabolic rate, limited intracellular peptide uptake, and proteome stability (Aguzzi and Rajendran, 2009; Bednarska et al., 2016). Furthermore, bacterial proteins are often less thermodynamically stable and have higher turnover rates, making them intrinsically more vulnerable to aggregation-mediated perturbation than mammalian proteins (Balch et al., 2008).

### APPs in therapeutic applications

The therapeutic feasibility of APPs has been demonstrated in vivo. In a murine methicillin-resistant *S. aureus* sepsis model, designed APPs successfully rescued infected animals without major host toxicity, validating the potential of proteostasis-targeting antimicrobials in systemic infection (Bednarska et al., 2016). Similarly, APPs based on redundant APRs showed efficacy against multidrug-resistant Gram-negative pathogens (*E. coli*, *Acinetobacter baumannii*) in a bladder infection model, underscoring their broad applicability (Khodaparast et al., 2018).

A key advantage of APPs lies in their multi-target mechanism: by inducing aggregation of multiple essential proteins simultaneously, they bypass classical single-target resistance mechanisms and reduce the likelihood of rapid resistance emergence. Moreover, the rapid bactericidal kinetics of APPs often surpass conventional antibiotics, reflecting the catastrophic impact of proteostasis collapse (Khodaparast et al., 2018). While the ability of APPs to trigger protein aggregation raises theoretical concerns about unintended aggregation of host proteins, current in vivo data indicate minimal off-target toxicity, suggesting that rational design and sequence specificity may provide sufficient safety margins in therapeutic contexts (Bednarska et al., 2016; Khodaparast et al., 2018). To summarize key contributions in the field, Table 3 highlights representative APP studies, their design principles, bacterial targets, and therapeutic outcomes. This comparison underscores how APPs, despite differences in origin or structural design, converge on the same mechanistic principle of proteostasis disruption as a bactericidal strategy.

## Targeting Proteolysis

Proteostasis maintenance is indispensable in the cytoplasmic environment, where numerous biological reactions crucial for bacterial survival occur. Within this space, specific ATP-dependent proteases such as Lon, HslUV, and ClpP serve as key components of the bacterial proteolytic machinery, ensuring the timely degradation of misfolded, damaged, or regulatory proteins. Disruption of these proteases offers an opportunity to impair bacterial viability by inducing proteolytic imbalance and proteostasis collapse.

### Lon protease

Among these systems, Lon has been the most extensively studied, with several inhibitors identified to date. For example, a rescreening of FDA-approved compounds identified nafcillin and diosmin as strong binders to *Salmonella* Typhimurium Lon protease, which effectively inhibited bacterial growth when co-administered with ceftazidime or ciprofloxacin (Narimisa et al., 2024). Similarly, MG262, a peptidyl boronate-based Lon inhibitor, reduced the proteolytic activity of *S*. Typhimurium Lon in an ATP-dependent manner (Frase et al., 2006). Another peptidyl boronate derivative, Molecule 11 (Pyz-hArg-nptGly-Leu-B(OH)₂), directly inhibited *E. coli* Lon protease, leading to abnormal cell division through SulA accumulation (Babin et al., 2019).

### ClpP protease

Among bacterial proteolytic systems, the Clp protease complex (ClpP and its associated unfoldases ClpA/ClpC) has gained particular attention as a tractable antibiotic target due to its substrate specificity and modular regulation. Adaptor proteins that deliver tagged substrates to the Clp complex differentiate it from other proteases, providing a unique therapeutic entry point.

A molecule that mimic adaptor proteins, such as cyclomarin A (CymA), binds to the N-terminus of *M. tuberculosis* ClpC1 unfoldase, stimulating the degradation of nascent polypeptides and causing cell death (Maurer et al., 2019). Moreover, several molecules trigger abnormal proteolysis by ATPase activity regulation of Clp unfoldases. Cyclic peptides like lassomycin and ecumicin act as ClpC1 activators, overstimulating ATPase activity and leading to proteotoxic collapse (Gao et al., 2015; Gavrish et al., 2014). In contrast, armeniaspirol from a Gram-positive bacterium *Streptomyces armeniacus* inhibit ATPase activity of Clp unfoldase and dysregulates cell division of bacterial cell. The armeniaspirol also acts on another cytoplasmic AAA+ protease HslUV system (Darnowski et al., 2022; Labana et al., 2021).

In addition to these modulators, several small molecules regulate the oligomerization or proteolytic activity of ClpP itself. For instance, ACPs (Activators of Self-Compartmentalizing Protease) bind the apical pocket of ClpP, enabling proteolysis without unfoldases (Barghash et al., 2025; Leung et al., 2011). Conversely, β-lactones derived from *Streptomyces coelicolor* irreversibly inhibit *M. tuberculosis* ClpP1/2 complex formation (Compton et al., 2013), while protocatechuic aldehyde (PCA) and coniferylaldehyde (CA) suppress *S. aureus* ClpP activity, with CA preventing degradation of ssrA-tagged peptides in an ATP-independent manner (Li et al., 2025a, 2025b).

One of the most intensively studied classes, ADEP (acyldepsipeptide antibiotics), dysregulates ClpP homeostasis through multiple mechanisms. ADEP forces the ClpP gate open, inducing uncontrolled proteolysis without Clp unfoldases, while simultaneously blocking ClpP–unfoldase complex formation (Kirstein et al., 2009; Malik and Brötz-Oesterhelt, 2017). This structural deregulation leads to the indiscriminate degradation of cytoplasmic proteins, driving lethal proteostasis collapse (Silber et al., 2020). Recent work further revealed a secondary ADEP mechanism, in which an ADEP-noninteracting ClpP copy enhances unfoldase-mediated proteolysis (Reinhardt et al., 2022). Natural products produced by ClpP-associated biosynthetic gene clusters (BGCs) in *Actinomycetales*, such as clipibicyclene, represent additional examples of this emerging antibiotic class (Culp et al., 2022).

### From proteolysis disruption to therapeutic innovation

Targeting proteolysis homeostasis offers a versatile strategy for antibacterial development by leveraging the essentiality and conservation of bacterial protease systems. While inhibitors can cause proteostasis collapse by preventing the removal of damaged proteins, activators or mimickers induce lethal over-degradation or dysregulation. Together, these dual approaches highlight how precise manipulation of bacterial proteolysis can be harnessed for therapeutic innovation. However, further research is needed to address off-target effects, species specificity, and potential compensatory mechanisms within the proteostasis network. The emergence of BacPROTAC systems (described in the following section) extends these concepts further—transforming the natural proteolysis machinery into a programmable antibacterial weapon.

## BacPROTAC for Targeted Protein Degradation in Bacteria

**Fig. 3.**
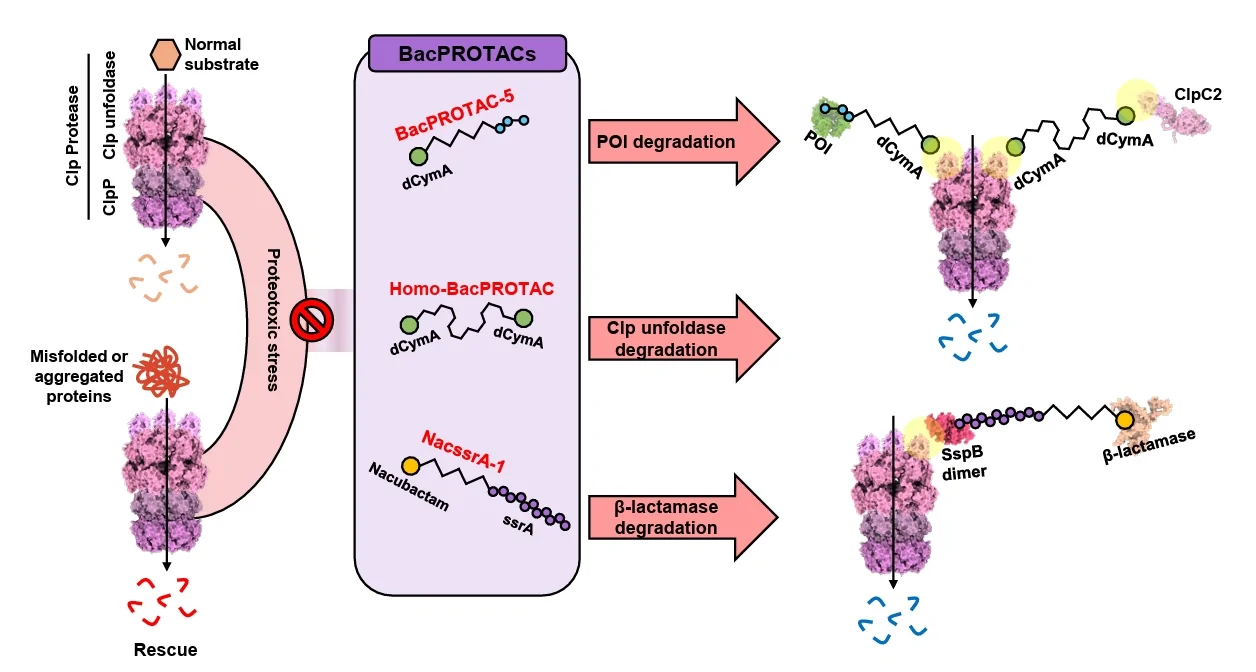
Dysregulation mechanisms of ClpP protease by BacPROTACs. BacPROTACs trigger abnormal proteolysis by ClpP protease hijacking. BacPROTAC-5 mediates degradation of the POI through bait-prey interaction and shorten physical distance between POI and ClpP protease. The POI-binding ligand in BacPROTAC-5 is shown in blue circles. Homo-BacPROTAC, a modified BacPROTAC, induces disassembly of ClpP protease by Clp unfoldase degradation. NacssrA-1, a Gram-negative bacteria-optimized BacPROTAC, causes degradation of β-lactamase by SspB adaptor system.

Recent advances in targeted protein degradation have extended beyond eukaryotic systems, paving the way for bacterial applications. Among these, BacPROTAC represents a pioneering approach that harnesses the cell’s own proteolytic machinery to selectively eliminate specific proteins. By enabling controlled degradation of essential or regulatory bacterial proteins, BacPROTAC offers new opportunities to modulate proteostasis, investigate protein function, and potentiate antibiotic efficacy (Fig. 3).

### Mechanistic basis of BacPROTAC function

Targeted protein degradation has recently emerged as a powerful antibacterial strategy for next-generation antibiotic development. In eukaryotic systems, PROTACs (PROteolysis Targeting Chimeras) induce selective degradation of target proteins through the ubiquitin–proteasome pathway (Espinoza-Chávez et al., 2023). In contrast, the bacterial version—BacPROTAC—adapts this concept to prokaryotic proteolytic machinery without ubiquitination (Trentini et al., 2016). Structurally, resting ClpC exists as a decamer, which converts into an active tetradecameric complex upon interaction with both substrate and BacPROTAC. This active form engages with the ClpP protease complex to trigger proteolysis.

The first-generation BacPROTAC (BacPROTAC-1) comprises a bifunctional molecule: one terminus carries a ClpC1-interactive moiety such as phosphorylated arginine (pArg), while the opposite end contains a target-binding ligand (Morreale et al., 2022). Due to the instability of pArg and its weak binding affinity toward *M. tuberculosis* ClpC1, later versions replaced it with cyclomarin A (CymA) derivatives, known adaptor-mimicking molecules of Clp unfoldases (Morreale et al., 2022). Chemical optimization of CymA yielded more stable and potent BacPROTACs—sCymA (BacPROTAC-2/3) and dCymA (BacPROTAC-4/5)—which display enhanced antibacterial activity against *M. tuberculosis*.

### Advances and applications of BacPROTAC in antibacterial design

The practical application of BacPROTACs as antibiotic agents relies on the identification of essential bacterial proteins suitable for degradation. In a *Mycobacterium smegmatis* model, screening through combined experimental and machine-learning approaches identified endogenous proteins with disordered termini as effective BacPROTAC targets (Won et al., 2024). Targeting these essential proteins not only induced bacterial cell death but also increased susceptibility to conventional antibiotics.

To further enhance BacPROTAC efficacy, researchers designed homo-dimerized BacPROTACs (Homo-BacPROTAC, HBP), in which two dCymA moieties are linked to directly recruit ClpC1 into self-degradative complexes (Junk et al., 2024). This approach allows rapid and efficient depletion of ClpC1 even at low concentrations and in intracellular infection conditions. However, the presence of ClpC2 in *M. tuberculosis* negatively regulates the ClpC1–ClpP1P2 complex formation, thereby attenuating BacPROTAC activity (Hoi et al., 2023). The HBP design, which targets both ClpC1 and ClpC2 via their Clp-repeat domains (CRDs), successfully reduces the levels of both proteins in *M. smegmatis* and restores degradation efficiency (Hoi et al., 2023).

As BacPROTAC is a recently developed antibacterial technology, bacterial resistance mechanism or toxicity (off-target effect) against to BacPROTAC treatment is not studied well so on. Although only one study showed natural resistance mechanism of ClpC2 in *M. tuberculosis*, HBP overcomes the ClpC2 disturbing by dual proteolysis of ClpC1 and ClpC2 (Hoi et al., 2023; Junk et al., 2024). HBP requires simultaneous engagement of its intended targets and exhibits highly low MIC value (Junk et al., 2024). Therefore, it is expected that HBP may impose less toxicity and fewer off-target effect than traditional antibacterial agents.

### Expanding BacPROTACs to Gram-negative systems

While initial BacPROTAC development was limited to Gram-positive bacteria, recent innovations have extended its applicability. NacssrA-1, a novel Gram-negative-specific BacPROTAC based on the ssrA-tagging degradation system, has demonstrated targeted proteolysis in these organisms (Nie et al., 2025). Moreover, peptide-based BacPROTACs such as CLIPPER (Clp-Interacting Peptidic Protein Eraser) introduce a modular protein–protein interaction approach. In this system, plasmid-encoded peptides include a substrate-binding anchor and a ClpX-binding bait connected by a flexible linker (Izert-Nowakowska et al., 2025). Notably, CLIPPERs targeting the essential chaperone GroEL in *E. coli* abolish bacterial growth by causing accumulation of misfolded and aggregated proteins, validating the feasibility of peptide-based BacPROTAC systems.

## Dormancy and Proteostasis-based Therapeutic Strategies

**Fig. 4.**
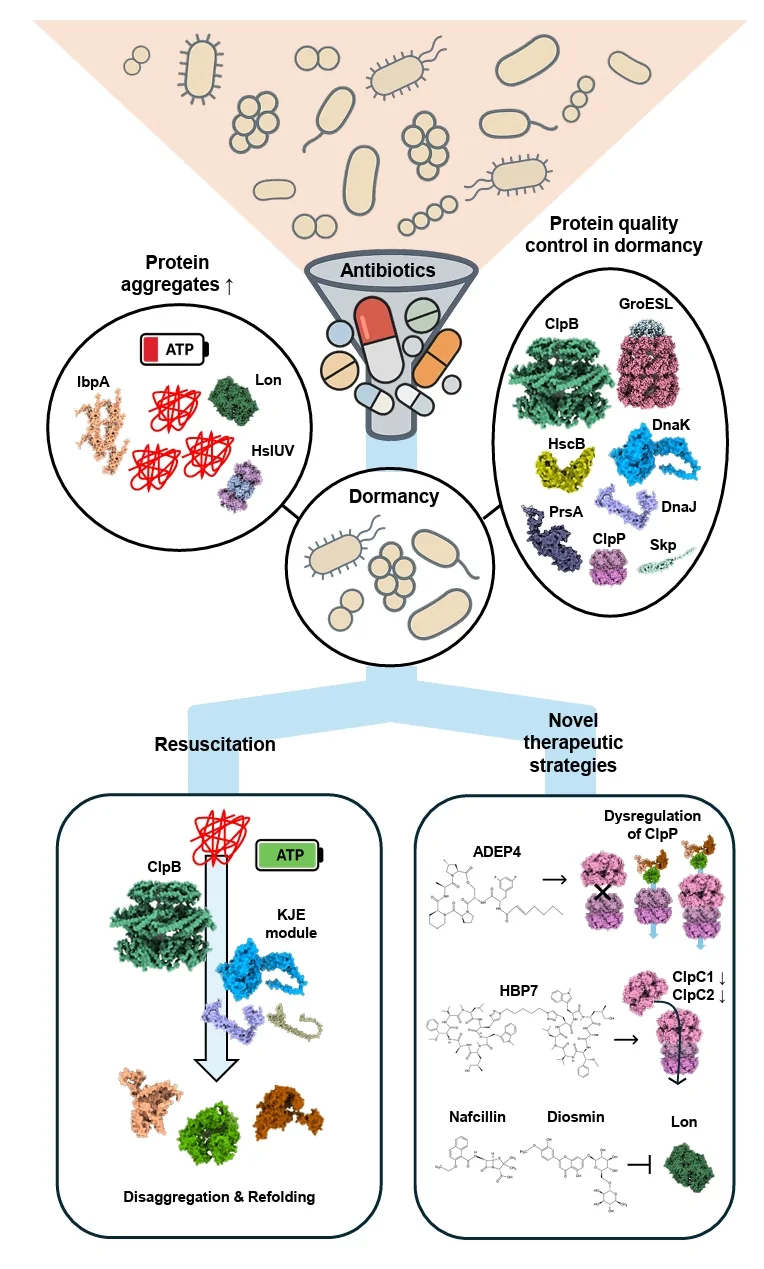
Flow of dormancy development in terms of proteostasis system. Dormant cells are emerged by incomplete killing by environmental stresses such as antibiotics. Under low ATP level, protein aggresomes are accumulated in the bacterial cell bodies through constant proteostasis system. As a consequence of protein aggresome accumulation, biological activities stop and the bacterial cells are much better defended against environmental stresses. After resolving of the environmental stresses, resuscitation occurs under high ATP level and the bacterial cells resume biological activities by diaggregation and refolding of protein aggresomes. However, proteostasis-targeting antibiotics such as ADEP, BacPROTAC, and nafcillin/diosmin can directly kill the dormant cells by dysregulation or degradation of molecular chaperones/proteases which are pivotal for protein aggresome formation.

Bacterial dormancy represents a survival strategy that allows cells to withstand environmental and antibiotic stresses by entering a reversible, low-metabolic state. This physiological shift is closely linked to proteostasis, as dormant cells rely on controlled protein quality management to maintain viability under stress. Understanding the interplay between dormancy and proteostasis not only reveals the molecular basis of bacterial persistence but also opens new avenues for therapeutic intervention aimed at reactivating or eradicating tolerant populations (Fig. 4).

### Protein aggregation and proteostasis in dormancy

Bacteria can transiently enter a dormant state to evade antibiotic-induced killing, which includes shallow-dormant persister cells and deep-dormant VBNC cells (Dewachter et al., 2021; Salcedo-Sora and Kell, 2020). Dormancy provides a non-energy-consuming resistance mechanism against diverse antibiotics, independent of genetic mutations or resistance pathways (Fang and Allison, 2023). Clinically relevant pathogens, including the ESKAPE group, utilize dormancy as a common survival strategy, and statistical analyses suggest persister emergence spans a broad range of antibiotic types (Salcedo-Sora and Kell, 2020; Van den Bergh et al., 2017). Understanding and therapeutically controlling dormant cells is therefore critical for public health.

Environmental stresses induce intracellular protein aggregation, which, although toxic in actively growing cells, appears to regulate dormancy depth in persister and VBNC cells. During dormancy, endogenous proteins progressively aggregate, forming mature aggresomes that can later be resolved by chaperones and proteases, such as IbpA, Lon, and HslU, to allow regrowth after stress relief (Dewachter et al., 2021; Pu et al., 2019). Conversely, modulation of aggregation—via heat shock, chemical triggers, or inhibition of protein synthesis—can alter persister and VBNC formation. ATP depletion further enhances aggregation by limiting the activity of ATP-dependent disaggregation systems, deepening dormancy (Dewachter et al., 2021). Notably, depletion of disaggregation systems like ClpB and DnaK delays resuscitation, underscoring the dependence of dormancy recovery on proteostasis.

### Proteostasis machinery in dormant cells

Proteomic and transcriptomic analyses indicate that proteostasis systems remain active, and in some cases upregulated, in dormant cells. Chaperones (e.g., ClpB, DnaK, DnaJ, and GroEL) and proteases (e.g., Lon, ClpX, and HslU) are maintained in *E. coli* persisters and *Vibrio cholerae* VBNC cells, supporting cellular integrity and eventual resuscitation (Debnath and Miyoshi, 2021; Radzikowski et al., 2016; Semanjski et al., 2021). Gram-positive persisters such as *S. aureus* also show upregulation of chaperones and proteases under antibiotic-induced stress (Liu et al., 2024b, 2024a). Functional proteostasis machinery is crucial not only for survival in dormancy but also for resuscitation after stress relief, as shown by delayed recovery following depletion of Fe-S cluster chaperone HscB or Lon protease (Mohiuddin et al., 2022; Spanka et al., 2019).

### Proteostasis-targeted therapeutic strategies

Given the essential role of proteostasis in dormancy, chaperones and proteases are emerging as novel therapeutic targets. ClpP and Lon proteases, key regulators of protein quality control and toxin-antitoxin systems, can be pharmacologically manipulated to disrupt dormancy (Petkov et al., 2023). ADEP4, a semisynthetic ClpP activator, induces uncontrolled proteolysis in *S. aureus* persisters, degrading hundreds of endogenous proteins even under ATP-limited conditions and enhancing susceptibility to rifampicin in biofilms (Conlon et al., 2013). Similarly, Lon inhibitors such as Pyz-hArg-nptGly-Leu-B(OH)₂, nafcillin, and diosmin reduce persister formation and potentiate antibiotic activity (Babin et al., 2019; Narimisa et al., 2024). Targeted protein degradation strategies, including HBP-mediated BacPROTAC systems, further extend efficacy against ATP-downregulated persister-like cells (Hoi et al., 2023). Together, these findings highlight that modulation of proteostasis machinery offers a promising approach for eliminating dormant bacterial populations.

## Conclusion

Bacterial proteostasis, maintained by chaperones and proteases, plays a central role in survival under antibiotic stress and during dormancy. Modulating these systems, through small-molecule inhibitors, BacPROTAC-mediated targeted protein degradation, or synergistic chaperone inhibition, represents a versatile and mechanistically innovative approach to potentiate antibiotic efficacy. Proteostasis-targeted strategies not only disrupt actively growing bacteria but also effectively target dormant persister and VBNC cells, which are refractory to conventional treatments. Future research should focus on developing selective modulators for both Gram-positive and Gram-negative pathogens, integrating multi-omics insights to identify critical targets, and designing combinatorial regimens that maximize bactericidal synergy while minimizing resistance evolution. Collectively, these advances underscore the potential of proteostasis-centered interventions to extend the therapeutic lifespan of existing antibiotics and address the global challenge of antimicrobial resistance.

## Figures and Tables

**Table 1.** Compounds targeting chaperones and proteases, and their effects described in Fig. 1

Annotation in Fig. 1	Name	Target sites	Effects	References
**1**	Telaprevir	SBD	Inhibit ATPase and chaperone activities of DnaK by disrupting allosteric coupling via substrate-mimicking interaction with the SBD	Hosfelt et al. (2022)
	BI-88E3	SBD	Disrupt allosteric interaction within DnaK	Cellitti et al. (2009)
	BI-88D7			
	BI-88B12			
	Nα-[Tetradecanoyl-(4-aminomethylbenzoyl)]-l-isoleucine	SBD	Inhibit the DnaK-mediated catalysis of cis/trans isomerization	Liebscher et al. (2007)
	Drosocin	SBD and C-terminal region	Inhibit ATPase and chaperone activities of DnaK by disrupting allosteric coupling via substrate-mimicking interaction with the SBD	Kragol et al. (2001); Otvos et al. (2000)
	Pyrrhocoricin			
	Apidaecin 1a			
	Bac-7	SBD	Impair DnaK-mediated refolding of denatured proteins	Zahn et al. (2014)
	CHP-105	Unknown	Synergistic effect with levofloxacin via DnaK inhibition	Credito et al. (2009)
	PET-16	NBD	Bind to ADP-bound DnaK and inhibit DnaK-client interaction	Leu et al. (2014)
**2**	Compound 8	Unknown	Bactericidal activity against *Escherichia coli*	(Abdeen et al. (2016)
	Compound 18			
	Hydroxquinolines	Unknown	Inhibit GroEL/ES activity by binding to the apical domain via a noncanonical, non-hydrophobic interaction	Stevens et al. (2020)
	Nifuroxazide	Apical domain	Inhibit the GroEL/ES folding cycle through apical domain binding	
	Bis-sulfonamido-2-phenylbenzoxazole	Apical domain	Inhibit ring-ring interaction of GroEL, compound derived from sulfonamido-2-arylbenzoxazole	Godek et al. (2024)
	Mizoribine	Equatorial domain	Inhibit ATPase activity of GroEL	Itoh et al. (1999)
**3**	BX-2819	N-terminal domain (NTD)	Inhibit ATPase activity of HtpG	Carlson et al. (2024)
	HS-291		BX-2819 derivative binds N-terminal ATP-binding pocket of HtpG, light-activated, triggers ROS generation	
	Polymixn B		Inhibit HtpG chaperone function without affecting its ATPase activity	Minagawa et al. (2011)
**4**	Cu^2+^-anthracenyl terpyridine complex	FKBP domain	Inhibit SlyD PPIase activity	Kumar and Balbach (2017)
**5**	β-Lactone	Active site serine in ClpP	Form covalent bond with the catalytic serine of ClpP and inhibit its proteolytic activity	Böttcher and Sieber (2008)
	Phenyl esters			Xiao et al. (2025)
	Peptide boronic acids			Akopian et al. (2015)
	Clipibicyclene			Culp et al. (2022)
	PCA	G107, V88, I81 in ClpP	Inhibit ClpP proteolytic activity	Li et al. (2025a)
	CA	M31, G33 in ClpP	Inhibit ClpP proteolytic activity by binding to active site residues M31 and G33	
	Ameniaspirol	ClpXP, HslUV (ClpYQ) complex	Competitively inhibit ClpXP and HslUV (ClpYQ)	Labana et al. (2021)
	CymA	NTD of ClpC1	Induce formation of large ClpC1 supercomplexes and activate associated ClpP protease via N-terminal domain binding	Taylor et al. (2022); Vasudevan et al. (2013)
	Lassomycin		Stimulate ClpC1 ATPase activity while inhibiting associated ClpP proteolytic activity	Gavrish et al. (2014)
	Ecumicin		Stimulate ClpC1 ATPase activity while inhibiting associated ClpP proteolytic activity	Hong et al. (2023); Hosfelt et al. (2022)
**6**	Nafcillin	Proteolytic active site	Interact with the binding pocket of Lon protease via hydrogen bonding; not reported as antibiotics	Narimisa et al. (2024)
	Diosmin			
	MG262		Form covalent bond with catalytic serine of Lon protease and inhibit its proteolytic activity	Frase and Lee (2007)
	Molecule 11	Unknown	Inhibition of Lon protease proteolytic activity	Babin et al. (2019)
**7**	ACP	Apical pocket	Enhance ClpP proteolytic activity independent of ATPase	Barghash et al. (2025); Leung et al. (2011)
	ADEP	Junction of the ClpP subunits	Bind to ClpP tetradecamer and enhance its proteolytic activity	Gersch et al. (2015)

**Table 2.** Chaperone inhibitors that potentiate antibiotic efficacy by disrupting bacterial proteostasis

Chaperone target	Representative inhibitors	Antibiotic partners	Model organism(s)	Reported effect	References
DnaK/Hsp70	Telaprevir (HCV protease inhibitor, repurposed)	Kanamycin, Streptomycin, Rifampicin	*M. smegmatis, M. tuberculosis*	Lowered MIC_50_ of aminoglycosides; reduced rifampicin resistance frequency; enhanced growth inhibition under heat/proteotoxic stress	Hosfelt et al. (2022)
DnaK/Hsp70	Proline-rich antimicrobial peptides (PrAMPs; e.g., pyrrhocoricin, synthetic dimers)	β-Lactams, Quinolones	*E. coli, Salmonella *spp.	Synergistic killing via disruption of DnaK folding function; accumulation of proteotoxic stress	Kragol et al. (2001)
GroEL/ES	Hydroxybiphenylamide derivatives	Aminoglycosides (e.g., gentamicin)	*Staphylococcus aureus*	Impaired folding capacity; reduced biofilm survival; enhanced aminoglycoside bactericidal activity	Kunkle et al. (2018)

**Table 3.** Representative aggregation-prone peptides (APPs) and their antibacterial activity

Peptide ID	Target bacteria	Sequence feature	Aggregation morphology	Mammalian toxicity	References
C30	MRSA	APR^*^ + Arg flanks	Amyloid-like	Low	Bednarska et al. (2016)
C29	MRSA	APR + Arg flanks	Amyloid-like	Low	
Hit50	MRSA	APR + Arg flanks	Amorphous inclusion bodies	Low	
Multiple APRs (263 tested)	*S. aureus*, *E. faecalis*, MRSA, Vancomycin-resistant Enterococcus	Tandem APR repeats, charged gatekeepers	Amyloid and amorphous aggregation	Low	Khodaparast et al. (2018)

* APR; aggregation-prone region.
